# Pharmacokinetics and Safety Profile of Artesunate-Amodiaquine Coadministered with Antiretroviral Therapy in Malaria-Uninfected HIV-Positive Malawian Adults

**DOI:** 10.1128/AAC.00412-18

**Published:** 2018-06-26

**Authors:** Clifford G. Banda, Fraction Dzinjalamala, Mavuto Mukaka, Jane Mallewa, Victor Maiden, Dianne J. Terlouw, David G. Lalloo, Saye H. Khoo, Victor Mwapasa

**Affiliations:** a Malawi College of Medicine, Blantyre, Malawi; b Malawi-Liverpool-Wellcome Trust Clinical Research Programme, Blantyre, Malawi; c Oxford Centre for Tropical Medicine and Global Health, Oxford, United Kingdom; d Liverpool School of Tropical Medicine, Liverpool, United Kingdom; e University of Liverpool, Liverpool, United Kingdom; f Mahidol-Oxford Tropical Medicine Research Unit, Bangkok, Thailand

**Keywords:** amodiaquine, antiretroviral therapy, malaria, nevirapine, ritonavir-boosted lopinavir

## Abstract

There are limited data on the pharmacokinetic and safety profiles of artesunate-amodiaquine in human immnunodeficiency virus-infected (HIV^+^) individuals receiving antiretroviral therapy. In a two-step intensive sampling pharmacokinetic trial, we compared the area under the concentration-time curve from 0 to 28 days (AUC_0–28_) of an active metabolite of amodiaquine, desethylamodiaquine, and treatment-emergent adverse events between antiretroviral therapy-naive HIV^+^ adults and those taking nevirapine and ritonavir-boosted lopinavir-based antiretroviral therapy. In step 1, malaria-uninfected adults (*n* = 6/arm) received half the standard adult treatment regimen of artesunate-amodiaquine. In step 2, another cohort (*n* = 25/arm) received the full regimen. In step 1, there were no safety signals or significant differences in desethylamodiaquine AUC_0–28_ among participants in the ritonavir-boosted lopinavir, nevirapine, and antiretroviral therapy-naive arms. In step 2, compared with those in the antiretroviral therapy-naive arm, participants in the ritonavir-boosted lopinavir arm had 51% lower desethylamodiaquine AUC_0–28_, with the following geometric means (95% confidence intervals [CIs]): 23,822 (17,458 to 32,506) versus 48,617 (40,787 to 57,950) ng · h/ml (*P* < 0.001). No significant differences in AUC_0–28_ were observed between nevirapine and antiretroviral therapy-naive arms. Treatment-emergent transaminitis was higher in the nevirapine (20% [5/25]) than the antiretroviral therapy-naive (0.0% [0/25]) arm (risk difference, 20% [95% CI, 4.3 to 35.7]; *P* = 0.018). The ritonavir-boosted lopinavir antiretroviral regimen was associated with reduced desethylamodiaquine exposure, which may compromise artesunate-amodiaquine's efficacy. Coadministration of nevirapine and artesunate-amodiaquine may be associated with hepatoxicity.

## INTRODUCTION

Human immunodeficiency virus (HIV) and Plasmodium falciparum malaria infections are endemic in most regions in sub-Saharan Africa (SSA), and coinfections occur frequently. HIV infection increases susceptibility (1–3) and severity of falciparum malaria (4–6) and reduces the efficacy of antimalarial drugs (7). The World Health Organization (WHO) recommends initiation of triple antiretroviral therapy (ART) in HIV-positive (HIV^+^) individuals regardless of CD4 cell count (8). The recommended ART in SSA contain nonnucleoside reverse transcriptase inhibitors (NNRTIs), such as efavirenz (EFV) and nevirapine (NVP), or protease inhibitors (PIs), such as ritonavir-boosted lopinavir (LPV/r). The WHO also recommends artesunate-amodiaquine (AS-AQ) as one of the first-line treatments for uncomplicated malaria (9).

Individuals coinfected with HIV and malaria require concurrent treatment with artemisinin-based combination therapies (ACTs) and ART, potentially resulting in pharmacokinetic (PK) interactions (10). Drug information sheets for ACTs caution against concurrent use of ACTs and ART because NNRTIs or PIs and ACTs are metabolized by cytochrome P450 (CYP450) liver enzymes (particularly CYP3A4). NNRTIs such as nevirapine and efavirenz usually induce various CYP450 enzymes but are also substrates for CYP450 isoforms (CYP3A4) (11, 12). AQ is rapidly metabolized, mainly by CYP2C8 but also CYP3A4, to its metabolite, desethylamodiaquine (DESAQ), which is responsible for almost all the antimalarial effect (13, 14). This metabolite has a longer half-life (*t*_1/2_) and is eliminated slowly compared to AQ (13–19). Thus, coadministration of NNRTI-based ART with AS-AQ could reduce AQ and DESAQ blood concentrations, resulting in lower efficacy of AQ. Conversely, HIV protease inhibitors, particularly ritonavir, are potent inhibitors of the CYP3A4 isoform. Coadministration of protease inhibitor-based ART with AS-AQ could lead to elevated AQ and lower DESAQ concentrations, potentially resulting in toxicities or reduced AS-AQ efficacy (20).

To characterize the interactions between AS-AQ and ART, we compared the pharmacokinetic parameters (area under the concentration-time curve from 0 to 28 days [AUC_0–28_], maximum concentration [*C*_max_], time to maximum concentration [*T*_max_], and *t*_1/2_) of the longer-acting partner drug of AS-AQ, amodiaquine, and of its metabolite, DESAQ, and incidence of adverse events (AEs) in HIV^+^ adults taking AS-AQ plus NVP ART or LPV/r ART and those taking AS-AQ only in a parallel-design (two-step) study.

## RESULTS

### Characteristics of study participants.

In step 1, 18 participants were successfully enrolled and followed up for 28 days, including 1 subject who replaced a participant who was withdrawn following a protocol violation. In step 2, 75 were enrolled and successfully followed up for 28 days, including 2 who replaced those who were lost to follow-up.

Table S1 in the supplemental material shows baseline characteristics of participants who completed follow-up in steps 1 and 2. In both step 1 and step 2, the majority of participants in all study arms, except the step 1 ART-naive arm, were females. Participants in the LPV/r arm had a tendency toward higher alkaline phosphatase levels at baseline than those in the ART-naive arm. In step 2, participants in the LPV/r arm had a higher median age than those in the other study arms. The median duration on ART was longer in the LPV/r than in the NVP arm. All the participants in step 1 and the majority (80%) in step 2 were on co-trimoxazole prophylaxis.

### Pharmacokinetics of AQ and DESAQ and interactions with ART in step 1.

PK data were available for 17 of the 18 participants who completed follow-up in step 1. The excluded participant had unquantifiable drug or metabolite concentrations at nearly all follow-up time points. AQ concentrations were well below the high-performance liquid chromatography (HPLC) assay limit of quantification (LOQ; 25 ng/ml). Therefore, no formal comparisons of AQ PK parameters were performed across the study arms.

**FIG 1:**
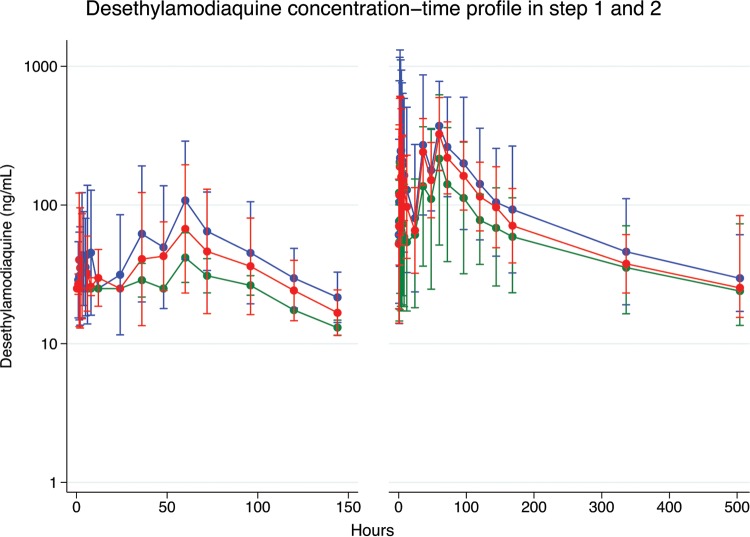
Desethylamodiaquine concentration-time profile (semilog scale) in step 1 (left; *n* = 17) and step 2 (right; *n* = 74) following oral administration of half and full standard artesunate-amodiaquine adult treatment courses, respectively, among HIV-infected ART-naive subjects (blue), those on nevirapine antiretroviral therapy (red), and those on ritonavir-boosted lopinavir-based (green) antiretroviral therapy. Concentrations below the limit of quantification are not included (resulting in observation time up to 144 h in step 1 and 504 h in step 2). Data are presented as means and 95% confidence intervals.

As shown in Table 1, the geometric mean (95% confidence interval [CI]) of DESAQ *C*_max_ was 60% lower in the LPV/r ART arm (42 [34 to 51] ng/ml) than in the ART-naive arm (106 [63 to 179] ng/ml; *P* = 0.006), while no significant difference in DESAQ AUC_0–28_ was observed between the LPV/r ART (4,128 [1,946 to 8,758] ng · h/ml) and the ART-naive (7,920 [5,034 to 12,459] ng · h/ml; *P* = 0.10) arms. The *C*_max_ values for DESAQ were similar between participants in the NVP and ART-naive arms. Similarly, no differences in mean AUC_0–28_ were observed between the NVP ART and ART-naive arms. As shown in the concentration-time plot in Fig. 1, the DESAQ concentration-time profile was notably lower in the LPV/r ART arm than in the ART-naive and NVP ART arms. There were no significant differences in half-life or *T*_max_ of DESAQ between the NVP and ART-naive arms or between the LPV/r and ART-naive arms.

**TABLE 1:** Desethylamodiaquine pharmacokinetic parameters for participants in step 1^*a*^

Parameter	Value for study group			Geometric mean ratio (*P* value)	
	ART naive (*n* = 5^*b*^)	NVP (*n* = 6)	LPV/r (*n* = 6)	NVP ART naive	LPV/r ART naive
AUC_0–28_ (h · ng/ml)	7,920 (5,034–12,459)	6,091 (3,096–11,983)	4,128 (1,946–8,758)	0.77 (0.465)	0.52 (0.100)
*C*_max_ (ng/ml)	106 (63–179)	75 (54–105)	42 (34–51)	0.71 (0.273)	0.40 (0.006)
*T*_max_ (h)	60 (36–60)	60 (3–60)	60 (36–60)	(0.562)^*c*^	(0.484)^*c*^
*t*_1/2_ (h)	59 (9–381)	88 (23–331)	75 (16–334)	1.49 (0.715)	1.27 (0.715)

a Values for PK parameters are presented as geometric means (95% confidence intervals) except for *T*_max_ values, which are reported as medians (ranges). *P* values were calculated using Wilcoxon rank sum test in Stata 15.0. ART, antiretroviral therapy; NVP, nevirapine-based ART; LPV/r, ritonavir-boosted lopinavir; AUC_0–28_, area under concentration-time curve from 0 h to 28 days; *C*_max_, maximal concentration; *T*_max_, time to reach maximal concentration; *t*_1/2_, drug elimination half-life.

b One participant did not have quantifiable DESAQ concentrations at nearly all follow-up time points and was excluded from analysis.

c *P* value only, calculated using Wilcoxon rank sum test.

### Safety assessment in step 1.

After AS-AQ administration, one participant in the NVP arm developed headache and chills, which resolved without any treatment and were judged as not related to the study drug. As shown in Table 2, treatment-emergent grade 3 or 4 neutropenia was observed in the NVP ART arm (50% [3/6]), LPV/r ART arm (33% [2/6]), and ART-naive arm (17% [1/6]). One participant in the arm with AS-AQ plus NVP had a car accident which was not thought to be related to the study drug.

**TABLE 2:** Summary of DAIDS grade 3 or 4 treatment-emergent adverse events in step 1

DAIDS (grade 3 or 4) treatment-emergent abnormality	No. (%) in treatment arm		
	AS-AQ without ART (*n* = 6)	AS-AQ + NVP (*n* = 6)	AS-AQ + LPV/r (*n* = 6)
Hematological events			
Anemia	0 (0)	1 (17)	0 (0)
Leukopenia	0 (0)	0 (0)	0 (0)
Lymphopenia	0 (0)	0 (0)	0 (0)
Neutropenia	1 (17)	3 (50)	2 (33)
Thrombocytopenia	0 (0)	0 (0)	1 (17)
Biochemical events			
Elevated ALT and AST	0 (0)	0 (0)	0 (0)
Raised creatinine	0 (0)	0 (0)	0 (0)
Cardiac events			
QTc prolongation	NA^*a*^	NA	NA

a NA, ECG assessment not conducted in step 1.

### Pharmacokinetics of DESAQ and interactions with ART in step 2.

In step 2, PK data were available for 74 of the 75 participants who completed follow-up. The excluded participant had unquantifiable drug or metabolite concentrations at nearly all follow-up time points. Similar to our observation in step 1, AQ concentrations in step 2 were well below the HPLC assay limit of quantification (25 ng/ml).

Table 3 shows that the geometric mean (95% CI) of DESAQ *C*_max_ was 45% lower in the LPV/r ART arm (248 [199, 310] ng/ml) than in the ART-naive arm (448 [74, 534] ng/ml; *P* < 0.001), while DESAQ AUC_0–28_ was 51% lower in the LPV/r ART arm (23,822 [17,458 to 32,506] ng · h/ml) than in the ART-naive arm (48,617 [40,787 to 57,950] ng · h/ml; *P* < 0.001). In contrast, there were no significant differences in AUC_0–28_ and *C*_max_ between the NVP and ART-naive arms. Also, there were no significant differences in DESAQ *T*_max_ among the ART-naive, LPV/r ART, and NVP ART study arms. DESAQ half-life and clearance were significantly shorter and faster, respectively, in the LPV/r ART arm than in the ART-naive arm.

**TABLE 3:** Desethylamodiaquine pharmacokinetic parameters for participants in step 2^*a*^

Parameter	Value for study group			Geometric mean ratio (*P* value)	
	ART naive (*n* = 25)	NVP (*n* = 25)	LPV/r (*n* = 24^*b*^)	NVP ART naive	LPV/r ART naive
AUC_0–28_ (h · ng/ml)	48,617 (40787–57,950)	43016 (38,300–48,313)	23,822 (17,458–32,506)	0.88 (0.308)	0.49 (0.0005)
*C*_max_ (ng/ml)	448 (374–534)	360 (322–403)	248 (199–310)	0.80 (0.067)	0.55 (0.0003)
*T*_max_ (h)	60 (1.5–96)	60 (3–60)	60 (2–72)	(0.887)^*c*^	(0.248)^*c*^
*t*_1/2_ (h)	166 (121–227)	234 (201–272)	90 (58–140)	1.41 (0.037)	0.54 (0.023)

a Values for PK parameters are presented as geometric means (95% confidence intervals) except for *T*_max_ values, which are reported as medians (ranges). *P* values were calculated using Wilcoxon rank sum test in Stata 15.0.

b One participant did not have quantifiable DESAQ concentrations at nearly all follow-up time points and was excluded from analysis.

c *P* value only compared using Wilcoxon rank sum test.

Figure 1 shows the concentration-time plot for DESAQ in the study arms. Similar to the findings in step 1, the DESAQ concentration-time profile in step 2 was notably lower in the LPV/r ART arm than in the ARV-naive arm.

### Day 7 plasma DESAQ levels by ART arm in step 2.

Compared with the geometric mean concentration (95% CI) of DESAQ at day 7 in the ART-naive arm (94 [73, 120] ng/ml), the concentration was 52% lower in the LPV/r arm (45 [29, 73] ng/ml; *P* = 0.011) and was 28% lower in the NVP arm (68 [57, 80] ng/ml; *P* = 0.092). However, there were no significant differences in the proportion of participants with day 7 DESAQ levels below 75 ng/ml (a threshold associated with 100% parasitological cure rate [19]) between the LPV/r arm (67% [14/21]) and the ART-naive arm (43% [9/21]; *P* = 0.215) or between the ART-naive arm and the NVP arm (56% [14/25]; *P* = 0.554).

### Safety assessment in step 2.

Overall, gastrointestinal symptoms (such as vomiting or diarrhea) or neurological symptoms (such as headache) were not reported following intake of AS-AQ in the different study arms. However, as shown in Table 4, there was a statistically nonsignificant trend toward higher incidence of grade 3 or 4 treatment-emergent neutropenia in the NVP arm (28.0% [7/25]) than in the ART-naive arm (16.0% [4/25]; *P* = 0.496). The incidence of grade 3 or 4 postdosing neutropenia was lower in the LPV/r arm (0.0% [0/25]; *P* = 0.110). The incidence of treatment-emergent grade 3 or 4 transaminitis (concurrent alanine aminotransferase [ALT] and aspartate transaminase [AST] elevations) was higher in the NVP arm (20% [5/25]) than in the ART-naive arm (0.0% [0/25]; risk difference, 20% [95% CI, 4.3, 35.7]; *P* = 0.018). Similar to the case with the ART-naive arm, there were no cases of treatment-emergent grade 3 or 4 transaminitis in the LPV/r arm. Two cases of QTc prolongation (change in QTc of >60 ms from baseline to *C*_max_) were detected in both the LPV/r ART arm (8.0%; *n* = 25) and the NVP ART arm (8.0%; *n* = 25), but none were detected in the ART-naive arm (0.0%; *n* = 25). No significant differences were found between either of the ART arms and the ART-naive arm (*P* = 0.490). These cases resolved spontaneously within 2 weeks of occurrence.

**TABLE 4:** Treatment-emergent DAIDS grade 3 or 4 abnormalities in step 2

DAIDS (grade 3 or 4) treatment-emergent abnormalities	No. (%) in treatment arm		
	AS-AQ without ART (*n* = 25)	AS-AQ + NVP (*n* = 25)	AS-AQ + LPV/r (*n* = 25)
Hematological events			
Anemia	1 (4)	0 (0)	0 (0)
Leukopenia	0 (0)	0 (0)	0 (0)
Lymphopenia	1 (4)	1 (4)	0 (0)
Neutropenia	4 (16)	7 (28)	0 (0)
Thrombocytopenia	0 (0)	2 (8)	0 (0)
Biochemical events			
Elevated ALT and AST	0 (0)	5 (20)	0 (0)
Raised creatinine	0 (0)	0 (0)	0 (0)
Cardiac events			
QTc prolongation	0 (0)	2 (8)	2 (8)

## DISCUSSION

In this study, we found that DESAQ AUC and *C*_max_ were significantly lower in the LPV/r arm than in the ART-naive arm, but no differences were observed in these PK parameters between the NVP and ART-naive arms. While AS-AQ appeared to be generally tolerated in all study arms, treatment-emergent transaminitis was more common in the NVP arm than in the ART-naive arm.

Our findings of insignificant differences in PK parameters of DESAQ between the ART-naive and NVP groups are in contrast with those from a previous Nigerian open-label parallel-arm PK study which found a lower DESAC AUC in HIV-infected adults on NVP-based ART than in ART-naive participants (21). These differences could be due to several reasons, including genetic differences in CYP450 isoenzymes of the study populations. Additional studies would be needed to explain the reasons for this discrepancy.

Although highly expressed in the liver, CYP family enzymes, especially CYP3A4 and CYP2C8, are expressed in the small intestinal epithelium and play an active role in the metabolism of drugs (22–24). Findings of significantly reduced DESAQ *C*_max_ in the LPV/r arm at the full standard dose in step 2 may partly be due to reduced CYP2C8-mediated gut or liver metabolism of AQ to DESAQ. This is plausible, as CYP2C8 is the main hepatic P450 isoform that clears AQ and catalyzes the formation of DESAQ (13, 25). Consequently, inhibition of CYP2C8 by its known potent inhibitors, LPV and ritonavir (10), is likely to account for the observed reduction in *C*_max_. Alternatively, the reduced DESAQ AUC in the LPV/r arm could be a result of rapid clearance of DESAQ in the LPV/r arm compared to that in the ART-naive arm. However, this increased clearance is inconsistent with the known inhibitory effects of LPV/r on CYP2C8 (25). DESAQ is eliminated through extrahepatic CYP1A1 and CYP1B1 (25, 26); any potential impact that LPV/r may have on clearance of DESAQ by CYP1A1 and CYP1B1 needs to be further evaluated.

Since DESAQ is responsible for nearly all the antimalarial effect of AQ (13, 14), it is likely that lower DESAQ exposure (reduced *C*_max_ and AUC at the full standard dose) in those taking LPV/r may result in lower treatment efficacy or prophylactic effect. Indeed, previous studies which administered amodiaquine base at a dosage of 10 mg/kg of body weight/day found that lower day 7 DESAQ concentrations were associated with an increased risk of treatment failure (14, 19). In a study by Stepniewska et al. (19), patients with day 7 DESAQ concentrations above 75 ng/ml achieved a 100% parasitological cure rate, while 60% (*n* = 5) of the participants who had day 7 DESAQ concentrations of below 75 ng/ml had PCR-confirmed recrudescent parasitemia. The daily and total amodiaquine doses received by participants in step 2 (9.5 mg/kg/day and 28.5 mg/kg, respectively) fall within the middle of the WHO's therapeutic dose range of 7.5 to 15 mg/kg/day for amodiaquine (9, 14, 27). The higher frequency of participants below the 75-ng/ml level in the LPV/r arm suggests that in this population, the current dosage of AS-AQ may likely result in treatment failure or recurrent malaria infections.

Our finding of a higher incidence of neutropenia in the NVP ART arm than in the ART-naive arm is consistent with results from a previous Ugandan study which found an increased risk of neutropenia in children receiving AQ-AS and ART (20). Although blood levels of AQ and AS were not measured in the Ugandan study, the observed cases of neutropenia could have been due to high AQ or DESAQ levels. NVP has been associated with granulocytopenia as a marker of hypersensitivity (28). Any potential synergistic role of AQ and NVP in causing neutropenia or other hematological abnormalities requires further study. Additionally, administration of AS-AQ in our study was associated with transient liver function abnormalities, especially in people taking NVP-based ART. This finding is similar to significant increases in liver transaminase levels observed in a previous study when AS-AQ was coadministered with an NNRTI (efavirenz) (29). NVP is independently associated with hepatotoxicity (30, 31), as is AQ (32, 33). Thus, combining these drugs may have an additive hepatotoxic effect. The observed cases of transaminitis in the NVP arm could have been due to an increase in NVP concentrations following coadministration with AQ or a result of a synergistic effect of NVP and AQ, as previously found among individuals taking an NNRTI (efavirenz) and AQ (29). Since we did not measure NVP concentrations, we were unable to ascertain the pharmacokinetic changes in steady-state concentrations of NVP after administration of AQ and the impact this may have had on incidence of transaminitis. Despite the fact that hematological and hepatic abnormalities found in our study were not clinically significant and did not persist beyond 2 weeks, our findings suggest that caution should be exercised when coadministering AS-AQ and NVP or the need for careful monitoring of liver function and hematological changes in malaria-infected HIV^+^ patients taking AS-AQ, particularly those taking AS-AQ plus NVP.

The present study was not adequately powered to detect adverse events such as cardiac toxicity. In our study, AQ levels were below the HPLC assay limit of quantification, possibly due to lack of sensitivity of the assay in detecting very low plasma drug concentrations. Although this study was not aimed at examining dose proportionality between the two steps, the inability to observe this and to detect significant differences in PK parameters across arms and between steps may have been due to a very small sample size in step 1 relative to step 2 and the use of the parallel-arm design, which is more prone to effects of interindividual anthropometric and genetic variations than a crossover design. Genetic polymorphisms in CYP450 isoenzymes may have contributed to wide interquartile ranges of DESAQ PK parameters observed within each study arm. However, our study sample size is unlikely to have missed large (>2-fold) clinically important differences in AUC across the study arms. Future studies should explore dose linearity when AS-AQ is administered with antiretroviral drugs, assess the effect of genetic polymorphisms on the pharmacokinetics of DESAQ, quantify any changes in plasma ART levels when coadministered with antimalarial drugs, and explore any potential impact of artesunate on the metabolism of amodiaquine when coadministered with antiretroviral drugs.

In conclusion, this study found significant PK interactions between LPV/r and AS-AQ and signals of transaminitis and neutropenic effects among those taking NVP and AS-AQ. The clinical therapeutic implications of these findings in malaria-infected individuals on ART need further evaluation.

## MATERIALS AND METHODS

### Study design.

We conducted an open-label, parallel-arm, pharmacokinetic (PK) trial at Queen Elizabeth Central Hospital, Malawi, from August 2010 to March 2013. The study was implemented in two steps.

In step 1 (*n* = 18) (PACTR2010030001871293), we administered half adult oral doses of AS-AQ (1 tablet of Coarsucam [Sanofi-Aventis], containing AS and AQ at 100 mg and 270 mg) at 0, 24, and 48 h, to HIV^+^ malaria-negative individuals in the following arms: (i) those on NVP-stavudine (d4T)-lamivudine (3TC), (ii) those on zidovudine (AZT)-3TC-tenofovir disoproxil fumarate (TDF)-LPV/r, and (iii) antiretroviral-naive individuals, who served as a control arm. Step 1 served as a safety evaluation step, checking for unexpected clinical toxicities or interactions.

In step 2 (*n* = 75) of the study (PACTR2010030001971409), after review of step 1 safety data by an independent data safety monitoring board (DSMB), full treatment doses of AS-AQ (2 tablets of Coarsucam [Sanofi-Aventis], each containing AS and AQ at 100 mg and 270 mg) were administered to additional HIV^+^ individuals in the same arms and at the same intervals as in step 1.

All doses of AS-AQ were administered with water only, as recommended by Sanofi-Aventis.

### Study population.

The target population for both steps included HIV^+^ male and nonpregnant female adults aged ≥18 years residing in Blantyre or the neighboring districts of Thyolo and Chiradzulu, Malawi. Individuals were eligible if they had been on NVP ART or LPV/r ART for ≥6 months and had CD4 cell counts of ≥250 cells/mm^3^. At the beginning of the study, HIV^+^ antiretroviral-naive individuals were eligible for recruitment into the study if they had a CD4 cell count of ≥250/mm^3^, but this cutoff point was increased to ≥350/mm^3^ when the new WHO criteria for ART initiation were implemented in Malawi in July 2011 (34). Other inclusion criteria were body weight of ≥40 kg and willingness to be admitted to the hospital for 3 days, to remain within the study sites, and to be contacted by phone or at home during the course of the study.

We excluded subjects who met any of the following criteria: (i) body mass index of ≤18.5 kg/m^2^, (ii) hemoglobin concentration of <8.5 g/dl, (iii) reported use of any antimalarial drugs within the preceding 4 weeks, (iv) reported hypersensitivity to any of the ACTs, (v) receipt of other drugs which are known inhibitors or inducers of P450 enzymes or P-glycoprotein (except co-trimoxazole prophylaxis), (vi) history of regular intake of alcohol (>twice/week) or tobacco (>3 times/week) or use of illicit drugs, (vii) history or evidence of preexisting liver, kidney, or heart disease, including conductive abnormalities on electrocardiographs (QTc interval of >450 ms in men or >470 ms in women), (viii) clinical and/or laboratory evidence of falciparum malaria, hepatitis B, pneumonia, tuberculosis, or bacteremia or laboratory evidence of potentially life-threatening disorders, and (ix) Karnofsky score of <80%.

### Sample size.

The sample size in step 1 was 6 for each of the three arms. This approach was based on standard practice in early PK studies of antimalarial drugs, which aims to safeguard the safety of study subjects and minimize the number of subjects who may be potentially exposed to harmful drug levels. The sample size for step 2 was 25 per arm, which gave at least 90% power to detect a 2-fold increase in the DESAQ AUC in any of the arms with AS-AQ plus ART, assuming a mean DESAQ AUC of 154 ng/ml/h (standard deviation of 150 ng/ml/h [2]) in the AS-AQ control arm, at the 5% significance level.

### Ethics.

The study conformed to the principles of the International Conference on Harmonization on Good Clinical Practice and was approved by the College of Medicine Research Ethics Committee (COMREC) in Malawi. Written informed consent to participate in the study was sought from potential participants.

### Screening and enrollment.

Research nurses and clinicians sought written informed consent from individuals to perform screening procedures, including physical medical and anthropometric assessment, electrocardiographs (ECGs), and blood tests to detect blood-borne infections and hematological, renal, or hepatic abnormalities. Based on the results from screening procedures which were available within 7 days, potential study participants were informed about their eligibility to participate in the study. Consenting study participants were reassessed by research nurses or clinicians to determine whether they still met all eligibility criteria, through repeat history taking and physical examination. Eligible participants were admitted to the hospital, and an indwelling cannula was inserted into a vein before their scheduled dose of ART and the first dose of the ACT. Approximately 1 h before the scheduled time of ART and ACT dosing, blood samples were collected for hematological and renal and liver function tests and random glucose testing.

### Blood sample collection and follow-up procedures.

While participants were hospitalized, blood samples for PK assays were collected in heparin Vacutainer tubes pretreatment and at the following posttreatment times: 0, 0.25, 0.5, 1, 1.5, 2, 3, 4, 5, 6, 8, 12, 24, 36, 48, 60, and 72 h. After discharge from the hospital, blood samples were taken at 4, 5, 6, 7, 14, 21, and 28 days. Immediately after collection, samples were spun in a refrigerated centrifuge and the separated plasma was temporarily frozen in liquid nitrogen before being transferred to a −80°C freezer until PK analyses.

Participants were monitored for 28 days after administration of the first study dose to detect clinical adverse events (AEs). Blood samples to detect hematological and renal and liver function abnormalities were collected at 12, 48, and 72 h and days 7, 14, 21, and 28. Participants were monitored for treatment-emergent AEs, defined as any clinical or subclinical abnormality which was absent before dosing with AS-AQ but emerged postdosing or a clinical or subclinical abnormality which was present before dosing with AS-AQ but worsened postdosing. Severity of AEs was graded using the DAIDS criteria (35). In addition, 12-lead ECGs were performed predosing, 2 h after the first dose, and 2 h after the last dose in step 2 to assess the Fridericia-corrected (36) QT interval.

### Pharmacokinetic assays.

Plasma samples were analyzed for AQ and DESAQ levels using a validated HPLC-UV assay adopted and transferred to Malawi-Liverpool Wellcome Trust Clinical Research Programme in Blantyre, Malawi, from the Liverpool School of Tropical Medicine. The PK laboratory in Blantyre participated in the WorldWide Antimalarial Resistance Network's (WWARN) External Quality Assurance program (37). Briefly, AQ/DESAQ and the internal standard (quinidine) were recovered from plasma using liquid extraction (diethyl/*tert*-butyl ether). The supernatant was evaporated to dryness in a vacuum concentrator at 25°C. The residue was redissolved in 200 μl of the reconstitution mobile phase, water-acetonitrile-triethylamine (85:15:1, vol/vol/vol; pH 3), and 75 μl was injected into the chromatograph (Agilent 1100). The optimum detection wavelength for each drug was 345 nm. The lower limit of quantification (LLOQ) of the HPLC-UV assay was 25 ng/ml for the drugs AQ and DESAQ. Extracted plasma PK samples were run in batches. Each batch run included a blank plasma extract, two sets of 8-concentration-level calibration standards, and quality controls (QCs) at three concentration levels: low, medium, and high (0.025, 1,500, and 3,000 ng/ml for AQ/DESAQ). For batch assay to pass the measured concentrations, at least 67% of the QC samples had to be within ±20% of their nominal value and at least one QC had to be acceptable at the LLOQ. In addition, 75% of each calibration curve's concentrations had to lie within ±20% and ±15% of the nominal concentration at the LLOQ or all other concentrations, respectively. The mean levels of interassay precision for low, medium, and high QCs were 15%, 9%, and 6%, respectively.

### Data analyses.

Plasma concentrations of AQ/DESAQ were analyzed using noncompartmental pharmacokinetic analysis (NCA), employing the trapezoidal rule with cubic splines. Observed AQ/DESAQ concentrations below LLOQ were treated as missing data except for the predose concentration, which was imputed to 0 if below LLOQ. For each study participant, the following PK parameters were computed: AUC_0–28_, maximum concentration [*C*_max_], time to maximum concentration [*T*_max_], and terminal elimination half-life [*t*_1/2_]. We used Stata 15.0 for the NCA and to compare PK parameters. The two-sample Wilcoxon rank sum (Mann-Whitney U test) was used to test any significant differences in PK parameters between each ACT/ART arm and the control arm (α = 0.05). Geometric means and their 95% confidence intervals are reported. Fisher's exact test was used to compare proportions of participants across the study groups with day 7 concentrations that were above a value known to predict treatment response by day 28, and of safety parameters across the different ACT/ART groups in comparison to the ART-naive group. Data summaries and graphics were all performed in Stata 15.0.

## Supplementary Material

Supplemental material
