# Supplementary material for: Pharmacokinetics and Safety Profile of Artesunate-Amodiaquine Coadministered with Antiretroviral Therapy in Malaria-Uninfected HIV-Positive Malawian Adults
Source: Antimicrob Agents Chemother. 2018 Jun 26;62(7):e00412-18. doi: 10.1128/AAC.00412-18 (PMC6021620; doi:10.1128/AAC.00412-18)
Supplement: Supplemental material [file supp_62_7_e00412-18__index.html]

Supplemental material 

# Pharmacokinetics and Safety Profile of Artesunate-Amodiaquine Coadministered with Antiretroviral Therapy in Malaria-Uninfected HIV-Positive Malawian Adults

## Supplemental material

- Supplemental file 1 -

  Table S1

  PDF, 63K
