# Supplementary material for: Pharmacokinetics and Safety Profile of Artesunate-Amodiaquine Coadministered with Antiretroviral Therapy in Malaria-Uninfected HIV-Positive Malawian Adults
Source: Antimicrob Agents Chemother. 2018 Jun 26;62(7):e00412-18. doi: 10.1128/AAC.00412-18 (PMC6021620; doi:10.1128/AAC.00412-18)
Supplement: Supplemental material [file AAC.00412-18_zac007187293s1.pdf]

Supplementary Table 1 – Baseline Characteristics for the participants with no protocol deviations/violations in the AS-AQ group for steps 1 and 2

Characteristic	Step 1				Step 2			
	NVP-based ART, N=6	LPV/r-based ART N=6	ART naive N=6	p-value	NVP-based ART, N=25	LPV/r-based ART N=25	ART naive N=25	p-value
Gender (% female)	6 (100)	6 (100)	3 (50)	0.074	20 (80.0)	18 (72.0)	16 (64.0)	0.452
Median age (range), years	32 (26-45)	39 (21-55)	31 (23-35)	0.138	37 (20-49)	50 (19-70)	35 (19-56)	0.016
Mean (SD) haemoglobin concentration, g/dL	11.6 (1.3)	12.4 (1.4)	13.0 (0.8)	0.216	13.7 (1.1)	12.3 (1.4)	13.3 (2.1)	0.006
Median (range) Body Mass Index, kg/m^2	21.1 (19.1-24.5)	23.4 (19.6-28.5)	24.2 (22.2-26.4)	0.079	21.4 (17.2-35.5)	22.3 (18.2-29.0)	20.7 (17.9-29.4)	0.646
Median (range) duration on ART, months	59.9 (16.4-79.6)	74.6 (38.9-83.6)	0 (0.0)	0.347	51.4 (11.4-89.4)	102.8 (24.5-135.1)	0 (0.0)	0.007
On Cotrimoxazole prophylaxis, n (%)	6 (100)	6 (100)	6 (100)	1.000	20 (80.0)	21 (84.0)	21 (84.0)	0.911
Median (range) ALT levels (IU/L)	18 (11-26)	14 (9-17)	15 (10-36)	0.304	21 (4-25)	16 (10-29)	18 (12-56)	0.117
ALT >ULN n (%)	0 (0.0)	0 (0.0)	0 (0.0)	1.000	2 (8.0)	2 (8.0)	3 (12.0)	0.854

Median (range) AST levels (IU/L)	27 (21-33)	22 (18-26)	31 (19-43)	0.288	26 (19-52)	24 (17-46)	26 (20-79)	0.388
ALT >ULN n (%)	0 (0.0)	0 (0.0)	0 (0.0)	1.000	5 (20.0)	2 (8.0)	5 (20.0)	0.520
Median (range) Alkaline Phosphatase levels, IU/L	58 (38-83)	97 (53-118)	56 (44-62)	0.033	63 (40-124	76.5 (31-240)	54 (31-77)	0.003
Median (range) Creatinine levels (umol/L)	61 (56-76)	57 (25-76)	64 (45-84)	0.863	61 (40-104)	61 (38-119)	70 (40-91)	0.070
Raised creatinine, n (%)	0 (0.0)	0 (0.0)	0 (0.0)	1.000	0 (0.0)	0 (0.0)	0 (0.0)	1.000
Any anaemia, n (%)	1 (16.7)	0 (0.0)	0 (0.0)	1.000	0 (0.0)	0 (0.0)	2 (0.0)	0.128
Any leucopenia, n (%)	0 (0.0)	0 (0.0)	0 (0.0)	1.000	2 (8.0)	0 (0.0)	0 (0.0)	0.128
Any neutropenia, n (%)	3 (50.0)	2 (33.3)	1 (16.7)	0.818	11 (44.0)	4 (16.0)	4 (16.0)	0.058
Any thrombocytopenia, n (%)	0 (0.0)	1 (16.7)	0 (0.0)	1.000	1 (4.0)	1 (4.0)	2 (4.0)	0.768
Median (range) CD4 cell count,, cells/UL	365 (257-415)	486 (257-839)	347 (254-692)	0.128	460 (261-952)	447 (300-1272)	378 (365-1202)	0.068
